# Microbial and Insect Gut-Mediated Polystyrene Microplastic Degradation for Environmental Remediation Applications

**DOI:** 10.3390/nano16130818

**Published:** 2026-07-02

**Authors:** Huy Loc Nguyen, Hong Minh Xuan Nguyen, Thi Bich Ngoc Nguyen

**Affiliations:** 1 Department of Engineering and Technology, Van Hien University, Ho Chi Minh City 72508, Vietnam; 2 Department of Chemical Engineering and Food Technology, Nong Lam University, Ho Chi Minh City 71308, Vietnam; nmxhong@hcmuaf.edu.vn; 3 Department of Water Management and Hydrological Sciences, Texas A&M University, College Station, TX 77843-2117, USA

**Keywords:** bioremediation, enzymatic depolymerization, insect gut microbiota, microbial degradation, polystyrene microplastics

## Abstract

Polystyrene (PS), particularly expanded polystyrene (EPS), is an environmentally significant commodity polymer that contributes substantially to secondary microplastic and nanoplastic pollution through environmental weathering and fragmentation. During aging, PS undergoes nano-scale physicochemical transformations, including chain scission, surface oxidation, and the formation of oxygen-containing functional groups, which profoundly influence its environmental fate, microbial colonization, and biodegradation behavior. Conventional remediation technologies remain energy-intensive and often fail to achieve complete mineralization, highlighting the need for sustainable and integrated remediation strategies. Recent studies have demonstrated that diverse microorganisms, including *Pseudomonas*, *Rhodococcus*, *Bacillus*, and *Exiguobacterium*, can colonize PS surfaces and initiate oxidative depolymerization through extracellular biofilm formation and oxidative enzymes such as styrene monooxygenase, laccases, and peroxidases. In parallel, insect-based systems, particularly *Tenebrio molitor* and *Zophobas morio*, provide unique biological platforms in which gut microbiota facilitate partial PS degradation and mineralization through synergistic host–microbe interactions. This review critically integrates recent advances in nano-scale PS transformation, microbial colonization, oxidative enzymatic pathways, insect gut-mediated biodegradation, and advanced analytical techniques used to characterize degradation processes. Emphasis is placed on nano–bio interactions and emerging nanotechnology-enabled remediation strategies, including engineered microbial consortia, biofilm-based bioreactors, and nanomaterial-assisted treatment systems. Finally, current limitations and future research priorities are discussed, including degradation kinetics, byproduct toxicity, standardized evaluation methods, and the integration of biological and nanomaterial-based approaches for scalable PS microplastic remediation.

## 1. Introduction

The global proliferation of plastics has created an unprecedented environmental burden, with microplastics now recognized as pervasive contaminants across terrestrial, freshwater, and marine ecosystems [1]. Although polyethylene (PE) and polypropylene (PP) dominate global plastic production, polystyrene (PS) remains an environmentally significant commodity polymer because of its widespread use in expanded polystyrene (EPS) packaging, disposable food containers, insulation materials, and consumer products [2]. Owing to its lightweight cellular structure, low mechanical strength, and extensive application in single-use products, EPS readily undergoes fragmentation during production, transportation, use, and disposal, making it a major source of secondary PS microplastics in the environment.

Environmental weathering progressively transforms PS through ultraviolet irradiation, thermo-oxidation, mechanical abrasion, and chemical oxidation, producing microplastics (<5 mm) and eventually nanoplastics [3]. This nano-scale transformation substantially alters the physicochemical properties of PS by increasing its specific surface area, surface free energy, and abundance of oxygen-containing functional groups. Compared with larger microplastics, nanoplastics exhibit greater mobility, enhanced contaminant adsorption, higher colloidal stability, and stronger interactions with microorganisms and biological membranes. Consequently, nano-scale transformation governs not only the environmental fate and toxicity of PS but also its subsequent microbial colonization, enzymatic accessibility, and biodegradation behavior.

The environmental and health implications of PS microplastics are increasingly concerning. These particles act as vectors for toxic additives and environmental pollutants, including heavy metals and persistent organic pollutants, and interact with biological systems across multiple trophic levels [4]. Their small size facilitates ingestion by organisms ranging from plankton to mammals, potentially leading to oxidative stress, inflammation, and disruption of metabolic processes [5]. Moreover, the inherent chemical stability of PS, characterized by a hydrophobic aromatic backbone composed of carbon–carbon bonds, renders it highly resistant to conventional degradation pathways [6]. These nano-scale physicochemical transformations also determine how PS interacts with biological systems. Oxidative weathering increases surface wettability and promotes the adsorption of dissolved organic matter and extracellular biomolecules, forming conditioning layers that facilitate microbial attachment and plastisphere development. Such nano–bio interactions strongly influence enzyme accessibility, microbial community succession, and the overall efficiency of biological degradation. Characterizing these transformations, therefore, requires advanced analytical techniques, including electron microscopy, spectroscopy, chromatography, and particle-size analysis, which collectively provide mechanistic insights into PS biodegradation.

Current strategies for managing PS waste are largely inadequate. Mechanical recycling is limited by contamination and material degradation, while chemical recycling methods often require high energy inputs and generate secondary pollutants [7]. Landfilling and incineration remain dominant disposal routes, contributing to environmental leakage and greenhouse gas emissions [8]. These limitations have driven increasing interest in biological degradation as a sustainable and potentially scalable alternative.

Biodegradation of synthetic polymers, particularly recalcitrant plastics, such as PS, has historically been considered negligible. However, emerging evidence suggests that diverse microorganisms possess the capacity to colonize plastic surfaces and initiate degradation processes [9]. Bacterial genera, including *Pseudomonas*, *Rhodococcus*, *Bacillus*, and *Exiguobacterium*, have been reported to adhere to PS surfaces and form biofilms, creating localized microenvironments conducive to enzymatic activity [10]. These microbial communities facilitate oxidative transformations that introduce functional groups into the polymer chain, thereby increasing susceptibility to further depolymerization.

Enzymes such as styrene monooxygenase, laccases, and peroxidases play key roles in the conversion of styrene units into more reactive intermediates [11,12]. Styrene monooxygenase catalyzes the epoxidation of styrene, forming styrene oxide, which can subsequently be metabolized into phenylacetic acid and integrated into central metabolic pathways [13]. Similarly, oxidative enzymes such as laccases and manganese peroxidases contribute to the generation of reactive oxygen species (ROS), facilitating chain scission and structural modification of the polymer matrix [14]. Despite these advances, the efficiency of microbial PS degradation remains relatively low, and the complete mineralization of the polymer is rarely achieved under natural conditions [15].

In parallel with microbial studies, insect-based systems have emerged as promising biological models for PS degradation. Notably, larvae of *Tenebrio molitor* (mealworms) and *Zophobas morio* (superworms) have demonstrated the ability to ingest and partially degrade PS materials [16]. These organisms rely on symbiotic gut microbiota to facilitate polymer breakdown, highlighting the importance of host–microbe interactions in plastic biodegradation. Studies have shown that disruption of gut microbiota significantly reduces degradation efficiency, confirming the microbial contribution to this process [17]. Within the insect gut, PS undergoes mechanical fragmentation, followed by microbial oxidation and assimilation, resulting in partial mineralization to CO_2_ and incorporation into biomass [18].

Another challenge lies in the scalability and practical implementation of biological degradation systems. While laboratory studies demonstrate promising results, translating these findings into industrial or environmental applications requires optimization of microbial consortia, reactor design, and process conditions [19]. Advances in synthetic biology and metabolic engineering offer potential solutions by enabling the design of microorganisms with enhanced degradation capabilities and tailored metabolic pathways [20].

Several review articles have previously summarized biological approaches for plastic degradation, including microbial degradation of synthetic polymers, enzymatic depolymerization, and insect-mediated plastic consumption [16,21]. These reviews have provided valuable overviews of plastic-degrading microorganisms, plastisphere formation, and the potential roles of larvae such as *Tenebrio molitor* and *Zophobas morio* in polystyrene transformation. However, relatively few reviews have integrated nano-scale PS transformation, microbial colonization, insect gut microbiota, oxidative enzymatic mechanisms, advanced nano-characterization, and emerging nanotechnology-enabled remediation within a single framework. As a result, the mechanistic links among PS surface aging, microbial colonization, oxidative enzyme activity, insect gut microbiota, degradation intermediates, and environmental engineering applications remain fragmented.

In addition, limited attention has been given to standardization of degradation metrics, byproduct toxicity, ecological safety, and the scalability of microbial or insect-derived systems for real-world PS microplastic remediation. Therefore, a focused synthesis is needed to integrate microbial and insect gut-mediated PS degradation mechanisms with emerging environmental applications and remaining translational challenges.

This review provides a comprehensive synthesis of microbial- and insect gut-mediated degradation of PS microplastics from a nanomaterials perspective. Particular emphasis is placed on nano-scale transformation during environmental aging, nano–bio interactions governing microbial colonization, oxidative enzymatic mechanisms, advanced analytical techniques for characterizing nano-scale structural changes, and emerging nanotechnology-enabled remediation strategies. By integrating microbiology, environmental engineering, and nanomaterials science, this review highlights current challenges and future opportunities for developing efficient and scalable approaches for PS microplastic remediation.

## 2. Review Methodology

This review was conducted using a structured and reproducible literature synthesis approach to capture current advances in microbial and insect gut-mediated degradation of PS microplastics. The methodology followed general principles of systematic and narrative reviews to ensure comprehensive coverage while allowing critical interpretation of emerging interdisciplinary findings.

### 2.1. Literature Search Strategy

A comprehensive literature search was performed across major scientific databases, including Web of Science, Scopus, PubMed, and Google Scholar. The search covered publications from 2000 to 2026, with particular emphasis on studies published after 2015 to reflect recent developments in plastic biodegradation.

Search queries combined keywords and Boolean operators, including: “*polystyrene microplastics*,” “*PS biodegradation*,” “*microbial degradation of polystyrene*,” “*styrene monooxygenase*,” “*laccase plastic degradation*,” “*insect gut microbiota plastic*,” “*Tenebrio molitor polystyrene*,” and “*Zophobas morio biodegradation*”. Additional searches incorporated terms related to enzymatic pathways, oxidative depolymerization, and environmental applications such as bioreactors and bioaugmentation.

Reference lists of key review papers and highly cited articles were manually screened to identify additional relevant studies. Forward citation tracking was also applied to capture newly published research building upon foundational work.

### 2.2. Data Extraction and Synthesis

Data extraction was performed systematically to capture key information from selected studies. Extracted parameters included microbial taxa, enzyme systems, experimental conditions (e.g., temperature, pH, and incubation time), analytical techniques, and degradation metrics such as mass loss, CO_2_ evolution, or intermediate formation. The data were then organized into thematic categories, including microbial degradation mechanisms, enzymatic pathways, insect gut-mediated processes, and environmental applications. A qualitative synthesis approach was applied to compare findings across studies, identify consistent mechanistic trends, and highlight discrepancies or gaps in current knowledge.

### 2.3. Quality Assessment and Limitations

The quality of included studies was evaluated based on methodological rigor, reproducibility, and strength of evidence for PS degradation. Studies employing multiple analytical techniques (e.g., combined spectroscopy and chromatography) and appropriate controls were considered more robust, with attention given to distinguishing true biodegradation from surface modification or physical fragmentation.

Despite efforts to ensure comprehensive coverage, several limitations should be acknowledged. Variability in experimental conditions, lack of standardized degradation metrics, and limited long-term studies complicate direct comparison across studies. Additionally, many investigations remain laboratory-based, with limited validation under real environmental conditions.

### 2.4. Scope and Framework of the Review

This review adopts an interdisciplinary framework integrating microbiology, enzymology, and environmental engineering perspectives. It focuses specifically on PS microplastics, while drawing mechanistic insights from related polymer systems where relevant. The synthesis emphasizes both fundamental mechanisms and translational potential, aiming to bridge the gap between laboratory discoveries and scalable environmental applications.

## 3. Sources, Environmental Aging, and Nano-Scale Transformation of Polystyrene Microplastics

### 3.1. Environmental Generation Pathways of Polystyrene Microplastics

PS microplastics originate from both primary and secondary sources, with the latter contributing the dominant fraction in environmental systems. Primary PS microplastics are intentionally manufactured at small sizes, such as industrial pellets or microspheres used in specialized applications, although their contribution is relatively minor compared to secondary sources [22]. Secondary PS microplastics are generated through the fragmentation of larger plastic debris, particularly expanded polystyrene (EPS) products widely used in packaging, insulation, and disposable food containers [23]. These materials are highly susceptible to environmental weathering processes, including ultraviolet (UV) radiation, thermo-oxidation, and mechanical abrasion, which induce polymer chain scission and surface embrittlement [24].

Photodegradation plays a particularly important role in PS fragmentation due to the polymer’s aromatic structure, which can absorb UV radiation and generate reactive intermediates such as free radicals [25]. These radicals initiate oxidative reactions that weaken the polymer backbone, leading to the formation of cracks and eventual fragmentation into microplastics. Mechanical forces, including wave action, wind erosion, and abrasion during waste handling, further accelerate this process, producing particles across a wide size distribution ranging from millimeters to nanometers [26].

In addition to environmental degradation, PS microplastics are released directly through anthropogenic activities. Industrial plastic processing facilities can emit PS particles through pellet loss, cutting residues, and wastewater discharge [27]. Urban environments contribute significantly through littering, improper waste disposal, and the breakdown of consumer products, while landfill systems act as long-term reservoirs where PS materials gradually degrade and release microplastics into the surrounding environments via leachate [28].

Collectively, these sources contribute to the continuous and diffuse input of PS microplastics into environmental compartments.

### 3.2. Transport Pathways in Aquatic and Terrestrial Systems

Once released into the environment, PS microplastics are redistributed through interconnected terrestrial, aquatic, and atmospheric pathways, resulting in widespread and persistent contamination [29]. In urban systems, stormwater runoff represents a dominant transport vector, mobilizing PS particles from roads, landfills, and impervious surfaces into drainage networks and ultimately into rivers, estuaries, and coastal waters [30]. Wastewater treatment plants (WWTPs) constitute another critical conduit, receiving microplastics from domestic, industrial, and municipal sources [31]. While conventional treatment processes, such as primary sedimentation, biological treatment, and filtration, can remove a substantial proportion of microplastics, their efficiency is highly dependent on particle size, density, and aggregation behavior, leading to incomplete removal and continued environmental release [32].

Microplastics that bypass treatment processes are discharged into receiving waters, where their environmental fate is governed by physicochemical properties and interactions with natural organic matter and biofilms. PS particles may remain suspended in the water column or undergo aggregation and sedimentation, contributing to accumulation in benthic environments [33]. Concurrently, a significant fraction of microplastics is retained in sewage sludge, which is frequently repurposed as agricultural fertilizer [34]. This practice introduces PS microplastics into soil ecosystems, where they can persist for extended periods, alter soil structure, and influence microbial community dynamics [35].

In addition to aquatic and terrestrial pathways, atmospheric transport has emerged as an important mechanism for the long-range dispersal of microplastics. Due to their low density and small size, PS particles can become resuspended and transported over considerable distances, facilitating their deposition in remote and previously unimpacted regions [36]. The integration of these transport pathways underscores the complex and dynamic distribution of PS microplastics across environmental compartments, highlighting the need for coordinated, cross-system management strategies to effectively mitigate their global spread.

### 3.3. Biofilm Formation and Environmental Aging

Environmental aging processes further modify the physicochemical behavior and environmental fate of PS microplastics. Prolonged exposure to ultraviolet (UV) radiation, temperature fluctuations, mechanical abrasion, and oxidative conditions induces significant alterations in surface morphology, chemical composition, and mechanical integrity [37]. These weathering processes promote chain scission and the formation of oxygen-containing functional groups such as carbonyls, hydroxyls, and peroxides, thereby increasing surface polarity and brittleness. As a result, PS particles become more fragmented, with increased surface roughness and porosity, which enhances their interaction with surrounding environmental matrices. Such transformations not only influence particle transport and aggregation behavior but also increase susceptibility to subsequent biological and chemical processes [38].

These physicochemical changes substantially facilitate microbial colonization, leading to the formation of biofilms on PS surfaces [39]. Biofilm development alters key surface properties, including density, hydrophobicity, and reactivity, often promoting aggregation with organic matter and influencing vertical distribution in aquatic systems [40]. In some cases, biofilm accumulation can increase particle density, causing initially buoyant PS microplastics to sink and accumulate in sediments, thereby expanding their environmental footprint [41].

**Figure 1:**
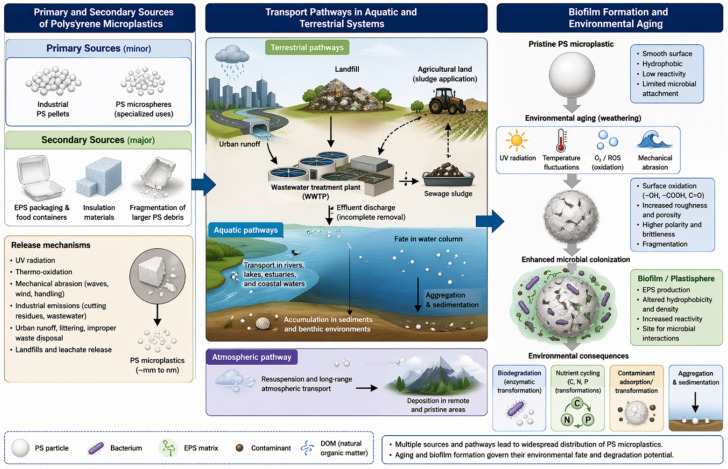
Integrated pathways of sources, transport, and environmental aging of polystyrene microplastics.

Figure 1 shows the schematic overview of PS microplastic sources, transport pathways, and environmental aging, highlighting fragmentation processes and plastisphere formation that govern their fate and behavior.

### 3.4. Nano-Scale Transformation of PS Microplastics

The environmental aging of PS extends beyond the formation of microplastics and ultimately generates nanoplastics, which exhibit physicochemical characteristics fundamentally different from those of larger particles. Progressive fragmentation is driven by the combined effects of ultraviolet irradiation, thermo-oxidation, mechanical abrasion, hydrolysis of oxidized fragments, and repeated environmental weathering. These processes induce polymer chain scission, reduce molecular weight, and produce particles ranging from several micrometers to hundreds of nanometers.

Particle-size reduction substantially increases the specific surface area of PS, resulting in higher surface energy and greater chemical reactivity. Simultaneously, oxidative weathering introduces oxygen-containing functional groups, including carbonyl, hydroxyl, and carboxyl moieties, which increase surface polarity and hydrophilicity. These physicochemical modifications influence aggregation behavior, colloidal stability, contaminant adsorption, and interactions with dissolved organic matter. Compared with larger microplastics, nanoplastics generally remain suspended in aquatic environments for longer periods and exhibit greater mobility through soils, sediments, and biological barriers.

Nano-scale transformation also alters the biological behavior of PS particles. The increased surface area promotes adsorption of proteins, natural organic matter, extracellular polymeric substances, and environmental contaminants, resulting in the formation of conditioning layers that facilitate microbial colonization. In addition, nanoplastics provide substantially greater contact area for oxidative enzymes and extracellular metabolites secreted by microorganisms, potentially accelerating the initial oxidative modification of the polymer surface. However, the same properties may also increase biological uptake by microorganisms, plants, and aquatic organisms, thereby enhancing ecological risks associated with nano-sized PS particles.

Advanced analytical techniques have significantly improved understanding of nano-scale PS transformation. Electron microscopy enables visualization of progressive surface erosion and fragmentation, while Fourier-transform infrared spectroscopy (FTIR), Raman spectroscopy, and X-ray photoelectron spectroscopy (XPS) identify oxidative functional groups generated during environmental weathering. Dynamic light scattering (DLS) and nanoparticle tracking analysis (NTA) provide information on particle-size distribution and colloidal behavior, whereas gel permeation chromatography (GPC) and pyrolysis-gas chromatography/mass spectrometry (Py-GC/MS) characterize reductions in molecular weight and identify degradation products. Collectively, these analytical approaches provide direct evidence linking environmental aging with nano-scale structural transformation and subsequent biodegradation. Because microbial degradation generally initiates at the particle surface, nano-scale transformation represents a critical intermediate stage connecting abiotic weathering and biological degradation. Therefore, future remediation strategies should consider environmental aging not merely as a fragmentation process but as a physicochemical preconditioning step that governs microbial colonization, enzyme accessibility, and overall biodegradation efficiency.

## 4. Microbial and Insect Gut-Mediated Degradation of Polystyrene Microplastics

### 4.1. Microbial Colonization and Degradation of Polystyrene

Despite their well-documented chemical recalcitrance, PS microplastics are increasingly recognized as substrates for microbial colonization across diverse environmental compartments. Upon introduction into natural systems, PS particles undergo rapid surface conditioning by dissolved organic matter, establishing a physicochemically altered interface that facilitates subsequent microbial attachment and community succession, a dynamic biofilm assemblage collectively termed the plastisphere [42]. This biofilm-mediated colonization represents a critical initiating step in the biodegradation cascade: it concentrates hydrolytic and oxidative enzymes at the polymer surface, sustains localized microenvironments conducive to reductive and oxidative reactions, and promotes intimate physical contact between microbial cells and the substrate. Bacterial genera including *Pseudomonas*, *Rhodococcus*, *Bacillus*, and *Exiguobacterium* have demonstrated a pronounced propensity for PS surface adhesion and the initiation of degradation-associated metabolic activity, underscoring the selectivity of plastisphere community assembly for organisms with polymer-interacting capabilities [43,44].

The interaction between microorganisms and PS is largely governed by surface physicochemical properties, including hydrophobicity, roughness, and surface charge [45]. PS is inherently hydrophobic, which can initially limit microbial adhesion; however, environmental weathering processes such as UV irradiation and oxidation introduce polar functional groups (e.g., carbonyl and hydroxyl groups), increasing surface wettability and facilitating microbial colonization [46]. Once attached to the PS surface, pioneer microorganisms secrete extracellular polymeric substances (EPS), initiating the development of a mature biofilm, commonly referred to as the plastisphere. EPS is not the biofilm itself but rather a hydrated extracellular matrix composed primarily of polysaccharides, proteins, extracellular DNA (eDNA), lipids, and other biopolymers that surround and embed microbial cells. This matrix provides structural integrity, enhances adhesion to the polymer surface, retains water and nutrients, protects microorganisms from environmental stress, and concentrates extracellular oxidative enzymes involved in polymer transformation [47].

Biofilm formation generally begins with the adsorption of dissolved organic matter and biomolecules onto the PS surface, forming a conditioning layer that modifies surface physicochemical properties and promotes microbial attachment. In protein-rich biological environments, this adsorbed biomolecular layer may also be described as a protein corona. Following initial attachment, microbial cells proliferate, secrete EPS, and establish a three-dimensional biofilm architecture in which multiple microbial species interact through metabolic cooperation. Such microbial consortia often exhibit complementary metabolic functions, whereby primary degraders initiate oxidative modification of PS while secondary microorganisms assimilate intermediate degradation products, thereby improving overall degradation efficiency and reducing the accumulation of potentially toxic intermediates.

Evidence for microbial degradation of PS is supported by multiple analytical approaches, including scanning electron microscopy (SEM), Fourier-transform infrared spectroscopy (FTIR), and gel permeation chromatography (GPC). Morphological changes such as surface pitting and cracking observed under SEM indicate microbial-induced physical damage, while FTIR analyses reveal the introduction of oxygen-containing functional groups, suggesting oxidative modification of the polymer chain [48]. Reductions in molecular weight further support the occurrence of chain scission processes. However, it is important to distinguish between true biodegradation and abiotic surface oxidation, as microbial activity often relies on pre-oxidized substrates to initiate further breakdown [49].

Microbial degradation of PS is generally slow and incomplete, primarily due to the high bond dissociation energy of the carbon–carbon backbone and the steric hindrance imposed by phenyl side groups. As a result, microbial metabolism typically targets low-molecular-weight intermediates rather than the intact polymer [50]. Styrene, a key monomer released during partial depolymerization, can be metabolized by certain bacteria via well-characterized pathways, including the side-chain oxygenation and ring-cleavage routes [51]. These pathways ultimately convert styrene into intermediates such as phenylacetic acid, which can enter central metabolic cycles such as the tricarboxylic acid (TCA) cycle.

**Figure 2:**
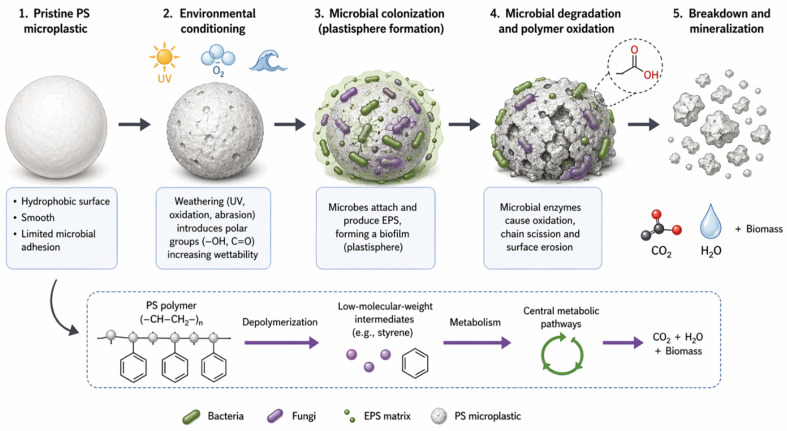
Schematic illustration of microbial colonization and biodegradation pathway of polystyrene microplastics.

Recent studies have also highlighted the importance of microbial consortia in enhancing PS degradation. Mixed microbial communities often outperform individual strains due to metabolic cooperation, where one organism initiates oxidation while others metabolize intermediate products. This division of labor can improve overall degradation efficiency and reduce the accumulation of potentially toxic intermediates [52]. Environmental isolates from soil, landfill, and marine systems have shown particularly promising results, suggesting that natural microbial communities may already possess adaptive mechanisms for interacting with synthetic polymers [53]. The stepwise process of microbial colonization and subsequent biodegradation of PS is illustrated in Figure 2.

Despite these advances, significant challenges remain in achieving efficient microbial degradation of PS. The rate of degradation is typically low, and complete mineralization is rarely observed under natural conditions [54]. Furthermore, variability in experimental conditions and analytical methods complicates comparisons across studies. Standardization of methodologies and the development of robust degradation metrics are, therefore, essential for advancing this field.

### 4.2. Enzymatic Mechanisms and Biochemical Pathways

**Figure 3:**
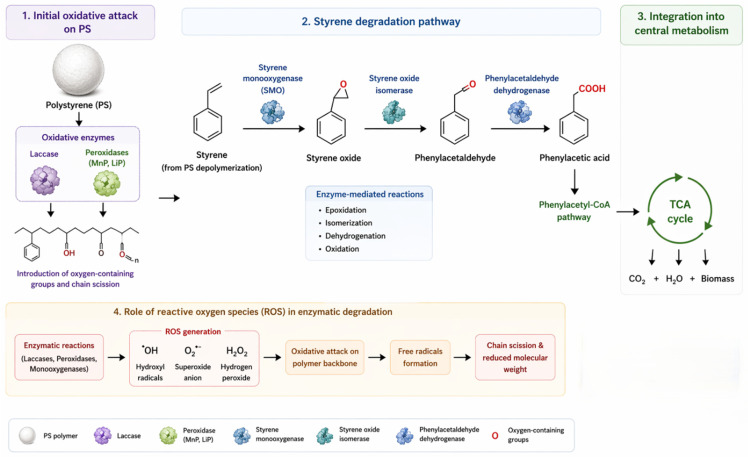
Schematic illustration of enzymatic mechanisms and biochemical pathways involved in polystyrene biodegradation.

The biodegradation of PS is primarily mediated through oxidative enzymatic processes rather than direct hydrolytic cleavage. This distinction arises from the chemical structure of PS, which lacks hydrolysable functional groups such as ester or amide bonds. Instead, degradation is initiated by enzymes capable of introducing oxygen into the polymer chain, thereby increasing its reactivity and susceptibility to subsequent breakdown [55]. The key enzymatic pathways and oxidative mechanisms involved in PS biodegradation are summarized in Figure 3.

One of the most extensively studied enzymatic systems involved in PS degradation is the styrene degradation pathway. Styrene monooxygenase (SMO) plays a central role in this pathway by catalyzing the epoxidation of styrene to styrene oxide, a reactive intermediate that can be further processed by styrene oxide isomerase and phenylacetaldehyde dehydrogenase [56]. These reactions ultimately yield phenylacetic acid, which is metabolized through the phenylacetyl-CoA pathway and integrated into central carbon metabolism. This pathway is well-documented in bacteria such as *Pseudomonas putida* and *Rhodococcus opacus*, which are capable of utilizing styrene as a carbon and energy source [57].

In addition to SMO, oxidative enzymes such as laccases and peroxidases have been implicated in the initial transformation of PS [58]. Laccases, which are multicopper oxidases, catalyze the oxidation of phenolic and non-phenolic substrates through electron transfer mechanisms, often generating reactive radicals that can induce polymer chain scission [59]. Similarly, peroxidases such as manganese peroxidase (MnP) and lignin peroxidase (LiP) utilize hydrogen peroxide to generate reactive intermediates capable of oxidizing aromatic structures [60]. These enzymes are particularly relevant in fungal systems, where ligninolytic pathways provide a model for degrading complex aromatic polymers.

Reactive oxygen species (ROS) play a critical role in enzymatic PS degradation by facilitating non-specific oxidative attacks on the polymer backbone. ROS such as hydroxyl radicals and superoxide anions can abstract hydrogen atoms from the polymer chain, leading to the formation of free radicals and subsequent chain cleavage. This process not only reduces polymer molecular weight but also introduces functional groups that enhance microbial accessibility [61]. The interplay between enzymatic activity and ROS generation is therefore a key factor in determining degradation efficiency.

Recent advances in omics technologies have provided deeper insights into the genetic and metabolic basis of PS degradation. Genomic and transcriptomic analyses have identified gene clusters associated with styrene metabolism, as well as regulatory networks that control enzyme expression in response to substrate availability [62]. Proteomic studies have further revealed the presence of extracellular enzymes and transport proteins involved in substrate uptake and metabolism [63]. Wang et al. (2024) investigated the role of gut microorganisms in polystyrene (PS) biodegradation by *Tenebrio molitor* larvae using selective antibiotics targeting Gram-negative, Gram-positive bacteria, and fungi [64]. Antibiotic treatments reduced PS consumption and degradation rates compared to the control, with the strongest inhibition observed for antifungal treatment. Microbial counts significantly decreased under all antibiotic conditions, confirming their role in degradation. Analytical techniques (DSC, TGA, FTIR) indicated oxidation and depolymerization of PS, though efficiency declined with microbial suppression. Results also suggest fungi, particularly *Candida*, along with bacterial groups such as *Enterobacteriaceae* and *Lactobacillus*, play key roles in PS biodegradation within the insect gut. These findings highlight the complexity of microbial degradation pathways and underscore the potential for metabolic engineering to enhance degradation capabilities.

Despite the identification of key enzymes, several challenges remain in elucidating the complete biochemical pathways of PS degradation. Many studies focus on intermediate compounds rather than the intact polymer, making it difficult to establish direct links between enzymatic activity and polymer breakdown [65]. Additionally, enzyme efficiency is often limited by substrate accessibility and environmental conditions, such as pH, temperature, and oxygen availability. Addressing these limitations will require integrated approaches combining biochemical characterization, systems biology, and process optimization.

### 4.3. Insect-Gut-Mediated Degradation and Symbiotic Interactions

**Figure 4:**
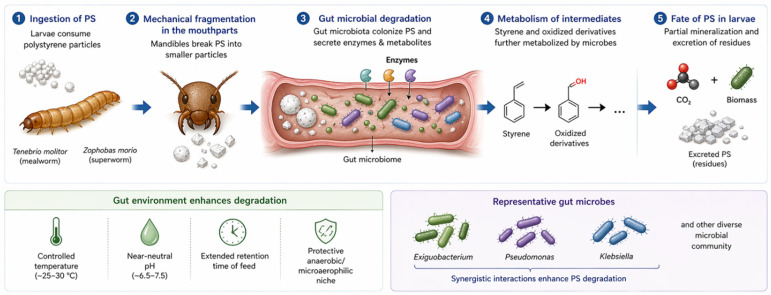
Insect Larvae-Microbiome system for polystyrene degradation.

In addition to microbial degradation in environmental matrices, insect-based systems have emerged as promising models for PS biodegradation (Figure 4).

Larvae of *Tenebrio molitor* and *Zophobas morio* have demonstrated the ability to ingest and partially degrade PS, providing a biologically tractable platform for studying host–microbe interactions in plastic degradation [18,66]. These organisms are capable of consuming PS as a sole or supplementary diet, with a portion of the ingested material being mineralized to CO_2_ and incorporated into biomass.

The degradation process in insect systems involves a combination of mechanical, chemical, and biological mechanisms. Mechanical fragmentation in the mandibles increases the surface area of PS particles, facilitating microbial access [67]. Within the gut, a complex microbiome interacts with the polymer, secreting enzymes and metabolites that contribute to its breakdown. Studies have shown that antibiotic treatment significantly reduces PS degradation rates, confirming the essential role of gut microbiota in this process [18].

The insect gut provides a unique microenvironment that enhances degradation efficiency. Factors such as controlled temperature, near-neutral pH, and extended retention time create favorable conditions for enzymatic activity [68]. Moreover, the gut microbiome often consists of diverse microbial communities capable of synergistic interactions. For example, bacteria isolated from the guts of mealworms include species of *Exiguobacterium, Pseudomonas*, and *Klebsiella*, many of which are known for their metabolic versatility [69]. These microbes may collectively contribute to the depolymerization and assimilation of PS.

Metabolic studies have revealed that PS degradation in insect systems produces intermediates like those observed in microbial pathways, including styrene and its oxidized derivatives [70]. These compounds are further metabolized by gut microbes, leading to partial mineralization. However, the extent of mineralization is typically limited, with a significant fraction of the polymer being excreted as fragmented residues [71]. The environmental fate and toxicity of these residues remain important areas of investigation.

The potential application of insect-based systems for plastic waste management has attracted considerable attention. Insects offer advantages such as low cost, scalability, and the ability to process heterogeneous waste streams [72]. However, several challenges must be addressed before practical implementation. These include optimizing feeding conditions, ensuring the safety of insect-derived products, and managing potential ecological risks associated with large-scale insect cultivation.

Recent research has focused on isolating and characterizing gut microbes responsible for PS degradation, intending to develop engineered microbial systems. By transferring these microbes or their enzymes into controlled bioreactor environments, it may be possible to achieve more efficient and scalable degradation processes [73]. Advances in synthetic biology and microbiome engineering further support this approach, enabling the design of tailored microbial consortia with enhanced degradation capabilities [74]. Insect gut systems provide valuable insights into the biological degradation of PS and highlight the importance of symbiotic interactions in overcoming the limitations of individual microorganisms. Continued research in this area is expected to contribute to the development of innovative and sustainable solutions for managing PS microplastic pollution.

## 5. Factors Influencing Polystyrene Biodegradation

### 5.1. Polymer Physicochemical Properties

The biodegradation behavior of polystyrene (PS) microplastics is fundamentally governed by intrinsic polymer properties, including molecular weight, crystallinity, surface chemistry, and hydrophobicity [75]. PS possesses a chemically inert backbone composed of strong carbon–carbon bonds and pendant phenyl groups, which collectively confer high resistance to enzymatic attack [76]. The absence of hydrolysable functional groups further limits biodegradation, necessitating oxidative preconditioning before microbial assimilation can occur. High molecular weight polymers are particularly resistant, as enzymatic systems typically act on low-molecular-weight fractions or oxidized intermediates rather than intact chains [77].

Surface chemistry plays a pivotal role in determining microbial interactions with PS. Pristine PS surfaces are hydrophobic and relatively smooth, limiting microbial adhesion [78]. However, environmental weathering processes, including ultraviolet (UV) irradiation, thermal oxidation, and mechanical abrasion, introduce oxygen-containing functional groups such as carbonyls and hydroxyls [79]. These modifications increase surface polarity and wettability, thereby enhancing microbial attachment and enzymatic accessibility [80]. Surface roughness and porosity further facilitate colonization by providing physical anchoring points for biofilm formation. As degradation proceeds, the increase in surface area through fragmentation enhances enzyme–substrate interactions, creating a positive feedback loop that accelerates localized degradation processes [81].

Another critical factor is the presence of additives and co-contaminants. Commercial PS often contains stabilizers, plasticizers, and flame retardants that can influence degradation pathways. Some additives may enhance degradation by modifying surface properties, while others may inhibit microbial activity due to toxicity or interference with enzymatic systems [82]. Additionally, PS microplastics can adsorb environmental pollutants such as heavy metals and hydrophobic organic compounds, which may further alter microbial interactions and degradation efficiency [83]. These complexities highlight the importance of considering real-world PS compositions rather than idealized laboratory materials.

### 5.2. Microbial Community and Biological Factors

Microbial community structure and diversity are key determinants of PS biodegradation efficiency. Individual microbial strains often exhibit limited capability to degrade PS due to the complexity of the polymer structure; however, mixed microbial consortia can significantly enhance degradation through synergistic interactions [84]. In such systems, primary degraders initiate oxidative modifications of the polymer, while secondary consumers metabolize intermediate compounds, preventing their accumulation and reducing toxicity [85]. This cooperative metabolism is particularly important for recalcitrant polymers like PS, where multiple enzymatic steps are required for effective degradation.

The formation of biofilms on PS surfaces is a critical biological process that facilitates degradation. Biofilms consist of microbial cells embedded in extracellular polymeric substances (EPS), which enhance adhesion and create microenvironments conducive to enzymatic activity. Within these microenvironments, localized concentrations of enzymes and metabolites can significantly increase reaction rates compared to planktonic systems [86]. Furthermore, biofilms enable horizontal gene transfer and metabolic cooperation, potentially accelerating the evolution of degradation capabilities.

Enzymatic diversity within microbial communities also influences degradation outcomes. Microorganisms capable of producing oxidative enzymes such as monooxygenases, laccases, and peroxidases are particularly important for initiating PS degradation. The expression of these enzymes is often regulated by environmental conditions and substrate availability, highlighting the importance of adaptive responses in microbial systems [87]. Advances in metagenomics and transcriptomics have revealed the presence of previously uncharacterized genes associated with plastic degradation, suggesting that microbial communities may harbor untapped potential for PS biodegradation.

### 5.3. Environmental Conditions and External Drivers

Environmental conditions play a crucial role in modulating PS biodegradation processes. Temperature, pH, oxygen availability, and nutrient levels directly influence microbial metabolism and enzymatic activity. Optimal temperatures enhance enzyme kinetics and microbial growth, whereas extreme conditions can inhibit biological processes. Oxygen availability is particularly important for oxidative degradation pathways, as many key enzymes involved in PS breakdown require oxygen as a substrate or co-factor [88]. In anaerobic environments, degradation rates are typically reduced, although alternative metabolic pathways may still contribute to partial transformation.

Nutrient availability also affects degradation dynamics. The presence of readily metabolizable carbon sources can either stimulate microbial growth through co-metabolism or suppress PS degradation due to preferential substrate utilization [89]. This dual effect underscores the importance of carefully controlling nutrient conditions in engineered systems. Additionally, environmental stressors such as salinity, pressure, and the presence of competing microorganisms can influence community structure and degradation efficiency [90].

Pre-treatment strategies have emerged as effective approaches for enhancing PS biodegradation. Techniques such as UV irradiation, plasma treatment, and chemical oxidation can introduce functional groups into the polymer, reducing molecular weight and increasing susceptibility to microbial attack [91]. Nanomaterial-assisted strategies are emerging as complementary approaches to enhance plastic degradation and control microbial interactions. Functionalized metal–organic frameworks, such as ZIF-8-based nanocomposites, have demonstrated strong antimicrobial and surface-modifying properties that can influence biofilm dynamics and potentially improve degradation efficiency in engineered systems [92]. These methods can significantly accelerate subsequent biological degradation, particularly when integrated into hybrid treatment systems. Overall, the interplay between environmental conditions and polymer properties determines the feasibility and efficiency of PS biodegradation in both natural and engineered settings.

## 6. Environmental and Engineering Applications

### 6.1. Microbial Bioreactor Systems

**Figure 5:**
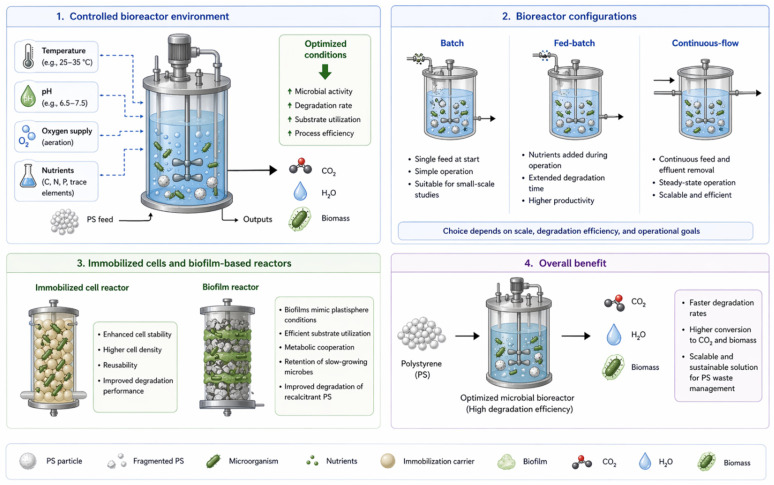
Schematic illustration of microbial bioreactor systems for polystyrene biodegradation.

A microbial bioreactor system represents one of the most promising approaches for translating PS biodegradation into practical applications (Figure 5).

These systems provide controlled environments in which key parameters such as temperature, pH, oxygen supply, and nutrient availability can be optimized to enhance microbial activity. Bioreactors can be designed as batch, fed-batch, or continuous-flow systems, depending on the desired operational scale and efficiency [93]. By maintaining optimal conditions, these systems can significantly improve degradation rates compared to natural environments.

The use of immobilized cells and biofilm-based reactors has gained particular attention. Immobilization techniques enhance microbial stability and allow for higher cell densities, leading to improved degradation performance [94]. Biofilm reactors, in particular, mimic natural plastisphere conditions, enabling efficient substrate utilization and metabolic cooperation. These systems also facilitate the retention of slow-growing microorganisms, which are often critical for degrading recalcitrant polymers like PS [85].

### 6.2. Bioaugmentation and Environmental Deployment

Bioaugmentation involves the deliberate introduction of specialized microorganisms into contaminated environments to enhance the biodegradation of polystyrene (PS) microplastics. This strategy has been explored in soils, composting systems, and wastewater treatment processes where indigenous microbial communities may lack sufficient enzymatic capacity to initiate or sustain PS degradation [95]. By introducing pre-selected PS-degrading strains or enriched microbial consortia, it is possible to accelerate oxidative depolymerization and subsequent assimilation of degradation intermediates. Studies have shown that bioaugmentation can significantly improve degradation rates, particularly when combined with co-metabolic substrates that stimulate microbial growth and enzyme expression [96].

Beyond laboratory-scale experiments, environmental deployment of bioaugmentation strategies is gaining traction in applied settings. For instance, landfill biocovers and composting facilities represent promising platforms where PS-degrading microbes can be introduced to enhance plastic breakdown under semi-controlled conditions [97]. In wastewater treatment plants, bioaugmentation can be integrated into existing activated sludge systems to target microplastic contaminants, potentially reducing PS accumulation in effluents and sludge [98]. Additionally, the use of carrier materials such as biochar, alginate beads, or porous scaffolds has been proposed to improve microbial survival, retention, and activity in complex environmental matrices. These carriers provide protective microhabitats and facilitate sustained enzymatic activity, thereby improving degradation efficiency [99].

Another emerging application is the development of biofilm-based remediation systems, where PS-degrading microorganisms are immobilized on surfaces or membranes to create stable and reusable treatment units [100]. Such systems can be deployed in contaminated waterways or industrial effluent streams, enabling continuous degradation under flow conditions. Furthermore, advances in microbial ecology have enabled the design of synthetic consortia tailored for specific environmental conditions, combining strains with complementary metabolic functions to enhance overall performance [85].

Despite these promising developments, several challenges remain for large-scale implementation. Environmental variability, including fluctuations in temperature, pH, and nutrient availability, can affect microbial activity and survival [101]. Competition with native microbial communities may also limit the establishment of introduced strains. Regulatory and ecological considerations, such as the potential spread of non-native or engineered organisms, must be carefully addressed. Future research should focus on improving the robustness of bioaugmentation systems, including the use of adaptive evolution, protective carriers, and real-time monitoring technologies to ensure consistent performance in diverse environments.

### 6.3. Insect-Based Bioconversion Systems

**Figure 6:**
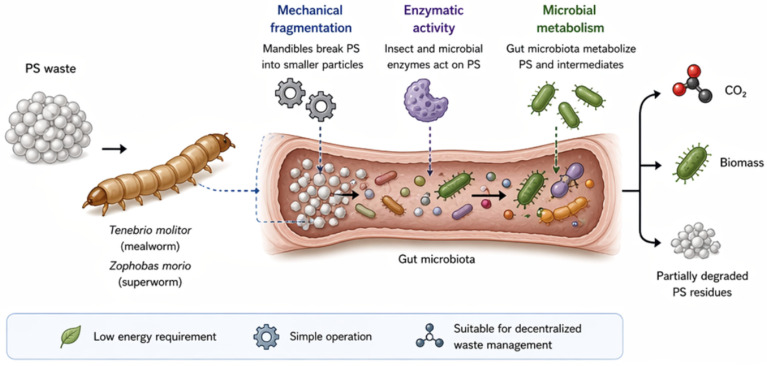
Mechanism of PS bioconversion in insect larvae.

Insect-based bioconversion systems have emerged as a novel and potentially scalable approach for managing PS waste (Figure 6).

Larvae of *Tenebrio molitor* (mealworms) and *Zophobas morio* (superworms) have demonstrated the ability to ingest and partially degrade PS, with their gut microbiota playing a central role in this process [18]. These systems operate through a combination of mechanical fragmentation, enzymatic activity, and microbial metabolism, converting PS into smaller fragments, CO_2_, and biomass [102]. The simplicity of these systems, coupled with their low energy requirements, makes them attractive for decentralized waste management applications [103]. Other organisms, including waxworms (*Galleria mellonella*), cockroaches, and potentially black soldier fly larvae (*Hermetia illucens*), have shown promise as biological models for plastic transformation, expanding the diversity of insect platforms available for biodegradation research [104]. Different species appear to contribute distinct advantages, such as high ingestion rates, prolonged gut retention, or diverse symbiotic microbiomes, which may influence degradation performance.

From an engineering perspective, insect-based bioconversion systems offer value beyond direct waste reduction by serving as biological models for designing engineered degradation platforms. One emerging application is the extraction or transfer of insect-gut microbial consortia into bioreactor systems, where controlled conditions can improve degradation kinetics compared to the native gut environment [105]. Another promising strategy is enzyme-inspired engineering, where oxidative enzymes identified in insect systems are isolated, immobilized, or recombinantly expressed for use in catalytic reactors or hybrid biofilm reactors. Insect digestion principles, including sequential fragmentation followed by microbial conversion, can also inspire multi-stage treatment trains that combine mechanical pretreatment, biocatalytic depolymerization, and downstream microbial mineralization [106]. Such concepts could be integrated into modular waste-treatment units, decentralized plastic management systems, or coupled biological upcycling processes for generating value-added intermediates.

Recent studies have explored the integration of insect-based systems into circular economy frameworks. For example, insect larvae can be used to process mixed plastic waste streams, reducing the need for pre-sorting and enabling the treatment of contaminated or low-value plastics [107]. The resulting insect biomass, rich in protein and lipids, has potential applications in animal feed, biofuel production, and other value-added products. However, the safety of such applications must be carefully evaluated, particularly with respect to the accumulation of plastic-derived contaminants and additives.

In addition to waste reduction, insect-based systems offer opportunities for biotechnological innovation. The gut microbiota of PS-consuming insects represents a rich source of novel enzymes and metabolic pathways that can be harnessed for engineered degradation systems [70]. Isolation and characterization of these microbes have already identified strains capable of degrading PS or its intermediates, providing a foundation for the development of microbial or enzymatic bioreactors [108]. Furthermore, advances in microbiome engineering may enable the optimization of gut microbial communities to enhance degradation efficiency and reduce byproduct toxicity.

Practical deployment of insect-based systems is also being explored in controlled rearing facilities, where environmental conditions such as temperature, humidity, and diet can be optimized to maximize degradation rates [109]. Modular insect-rearing units could be integrated into waste management infrastructures, particularly in regions lacking advanced recycling technologies [110]. Additionally, hybrid systems combining insect digestion with downstream microbial or chemical treatments may further improve overall degradation efficiency and enable more complete mineralization of PS [46].

Despite their potential, insect-based systems face several limitations. Degradation rates are still relatively slow, and a significant portion of ingested PS is excreted as partially degraded residues [111]. The long-term environmental impact of these residues remains unclear. Moreover, scaling up insect-based systems requires careful consideration of logistics, including feedstock supply, waste handling, and biosecurity [112]. Addressing these challenges will be essential for translating insect-based PS degradation from experimental models to practical applications. Table 1 summarizes the major environmental and engineering application strategies currently explored for biological degradation of polystyrene microplastics, including microbial bioreactor systems, bioaugmentation and hybrid treatment approaches, insect-gut bioconversion, and biological upcycling platforms.

Collectively, these strategies rely on oxidative depolymerization, microbial or symbiotic metabolism, and emerging engineered pathways to transform PS-derived carbon. While these approaches offer advantages such as scalability, enhanced degradation efficiency, and circular-economy potential, significant challenges remain, including slow degradation kinetics, incomplete mineralization, process optimization, and large-scale implementation. These limitations highlight the need for integrated treatment designs and further advances in microbial engineering and process development.

## 7. Challenges, Knowledge Gaps, and Future Perspectives

Although considerable progress has been made in understanding the biological degradation of PS microplastics, several critical challenges and knowledge gaps remain. One of the most significant limitations is the inherently slow rate of biodegradation. Compared to biodegradable polymers such as polyesters, PS degradation occurs over extended time scales, often requiring weeks to months under laboratory conditions [117]. These slow kinetics limit the practicality of biological approaches for large-scale environmental remediation and highlight the need for strategies to accelerate degradation processes.

Another major challenge is the incomplete understanding of degradation pathways and intermediate products. While key enzymes and metabolic routes have been identified, the transformation of PS into fully mineralized end-products remains poorly characterized. Intermediate compounds such as styrene, styrene oxide, and oligomeric fragments may accumulate during partial degradation and pose potential environmental and health risks [54]. Comprehensive identification and toxicity assessment of these byproducts are therefore essential for evaluating the sustainability of biodegradation strategies.

Standardization of experimental methodologies represents an additional gap in the field. Variability in experimental conditions, analytical techniques, and reporting metrics makes it difficult to compare results across studies. For example, degradation efficiency may be reported in terms of weight loss, molecular weight reduction, or CO_2_ evolution, each providing different insights into the degradation process [118]. Establishing standardized protocols and metrics would improve reproducibility and facilitate the development of predictive models for PS biodegradation.

The scalability of biological degradation systems also remains a significant barrier. While laboratory studies demonstrate proof-of-concept, translating these findings into industrial or environmental applications requires overcoming challenges related to reactor design, process optimization, and cost-effectiveness. Maintaining stable microbial communities and consistent degradation performance under variable environmental conditions is a key concern [119]. Advances in bioprocess engineering and systems biology are expected to play a critical role in addressing these challenges.

Future research should focus on integrating multidisciplinary approaches to enhance PS degradation. The application of omics technologies, including genomics, transcriptomics, and metabolomics, can provide deeper insights into microbial communities and metabolic pathways. These tools can be combined with machine learning and data-driven modeling to identify key determinants of degradation efficiency and guide the design of optimized systems [120]. Additionally, strategies for polystyrene (PS) microplastic management should integrate antimicrobial nanomaterials to control plastisphere dynamics. ZIF-8 and its derivatives nanocomposites have shown strong antibiofilm activity on food-contact surfaces [121,122]. Translating this concept to PS systems suggests that such materials could regulate microbial colonization on microplastics, suppress pathogenic biofilms, and potentially promote degradation-active communities. Incorporating ZIF-8-based nanomaterials into PS remediation frameworks offers a dual-function approach, combining biofilm control with enhanced biodegradation, and represents a promising direction for safer and more efficient microplastic management in environmental and food systems. Synthetic biology offers opportunities to engineer microorganisms with enhanced capabilities, including improved enzyme expression, substrate specificity, and tolerance to environmental stressors.

Another promising direction is the development of circular economy frameworks for plastic waste management [122]. Instead of focusing solely on degradation, future strategies may aim to convert PS into valuable products, such as biofuels, chemicals, or functional materials. This approach aligns with sustainability goals by transforming waste into resources and reducing reliance on fossil-based feedstocks. However, achieving this vision requires a deeper understanding of metabolic pathways and efficient integration with existing industrial processes.

While biological degradation of PS microplastics holds significant promise, its practical implementation requires addressing fundamental and applied challenges. Future research efforts should prioritize improving degradation efficiency, understanding environmental impacts, and developing scalable technologies. By bridging the gap between laboratory research and real-world application, biological approaches have the potential to contribute meaningfully to the global effort to mitigate plastic pollution.

## 8. Conclusions

Polystyrene (PS) microplastics represent one of the most persistent and challenging forms of plastic pollution due to their chemical-inert structure, hydrophobicity, and resistance to conventional degradation processes. This review highlights that, despite these constraints, emerging biological pathways, particularly those mediated by microorganisms and insect gut systems, offer promising avenues for PS transformation and partial mineralization. Microbial communities, including genera such as *Pseudomonas*, *Rhodococcus*, and *Bacillus*, have demonstrated the ability to colonize PS surfaces and initiate oxidative depolymerization, primarily through enzymes such as styrene monooxygenase, laccases, and peroxidases. These enzymatic processes convert PS into lower-molecular-weight intermediates that can be assimilated into central metabolic pathways, although complete mineralization remains limited under most conditions.

In parallel, insect-based systems, particularly those involving *Tenebrio molitor* and *Zophobas morio*, provide compelling models of synergistic degradation, where host–microbiome interactions facilitate polymer breakdown. The unique physicochemical environment of the insect gut enhances microbial activity and enables partial conversion of PS into CO_2_ and biomass. These findings underscore the importance of microbial consortia and cooperative metabolic networks in overcoming the intrinsic recalcitrance of PS. Furthermore, advances in omics technologies and microbial ecology have begun to unravel the complexity of these systems, revealing novel enzymes and pathways that may be harnessed for improved degradation performance.

Despite these advances, several critical challenges must be addressed before biological PS degradation can be translated into scalable and sustainable solutions. Degradation rates remain slow, and the accumulation of intermediate products such as styrene derivatives raises concerns regarding environmental toxicity and process efficiency. In addition, the lack of standardized methodologies and inconsistent reporting metrics across studies limits comparability and hinders the development of predictive models. From an engineering perspective, the design of robust and economically viable systems capable of operating under variable environmental conditions remains a significant barrier.

Future research should focus on integrating multidisciplinary approaches to enhance PS biodegradation. The application of synthetic biology and metabolic engineering offers opportunities to develop tailored microbial strains and consortia with improved enzymatic capabilities and stress tolerance. Coupling biological processes with physicochemical pre-treatment strategies, such as UV or plasma oxidation, may further enhance degradation efficiency by increasing polymer accessibility. Additionally, the incorporation of systems-level tools, including genomics, metabolomics, and machine learning, can facilitate the identification of key drivers of degradation and guide the optimization of engineered systems.

Importantly, the transition toward circular economy frameworks presents an opportunity to reframe PS waste not solely as an environmental liability but as a potential resource. Biological conversion pathways may enable the transformation of PS into value-added products, contributing to more sustainable material cycles. However, achieving this vision will require careful assessment of environmental risks, regulatory considerations, and lifecycle impacts. In conclusion, while microbial and insect gut-mediated degradation of PS microplastics remains in its early stages of development, it represents a rapidly evolving field with significant potential. Continued interdisciplinary research, combined with advances in biotechnology and process engineering, will be essential to overcome existing limitations and unlock the full potential of biological strategies for mitigating PS microplastic pollution.

## Figures and Tables

**Table 1:** Major environmental and engineering applications for the biological degradation of polystyrene microplastics.

Application Category	Core Mechanism	Advantages	Challenges/Future Needs
Microbial bioreactor and biofilm systems	Oxidative depolymerization, styrene metabolism, immobilized-cell degradation	Scalable, controlled operation, compatible with wastewater treatment	Improve degradation kinetics, reduce fouling, and achieve complete mineralization [46,113]
Bioaugmentation and hybrid treatment systems	Specialized degraders combined with oxidative or physical pre-treatment	Enhanced polymer accessibility and improved biodegradation efficiency	Optimize integrated treatment trains and validate field-scale performance [114].
Insect-gut bioconversion systems	Mechanical fragmentation coupled with gut microbiota-mediated transformation	Low energy demand and decentralized treatment potential	Address incomplete degradation, residue toxicity, and scale-up limitations [18,115].
Biological upcycling and engineered systems	Conversion of PS-derived intermediates into fuels or value-added chemicals	Supports circular economy and resource recovery	Improve product yields, metabolic engineering [116].

## Data Availability

No new data were created or analyzed in this review study. Data sharing does not apply to this article.
